# Phytochemical and Antioxidant Profile of Pitaya (*Hylocereus hybridum*) Fruits: Elucidation Through Chemical Fractionation

**DOI:** 10.1111/1750-3841.70502

**Published:** 2025-08-22

**Authors:** Noemi Gatti, Graziella Serio, Jaime Morante‐Carriel, Pietro Deusebio, Giulia Conti, Campo Eva, Moez Maghrebi, Carla Gentile, Giuseppe Mannino

**Affiliations:** ^1^ Department of Life Sciences and Systems Biology, Innovation Centre University of Turin Turin Italy; ^2^ Department of Biological, Chemical and Pharmaceutical Sciences and Technologies (STEBICEF) University of Palermo, Viale delle Scienze Palermo Italy; ^3^ Plant Proteomics and Functional Genomics Group, Department of Biochemistry and Molecular Biology and Soil and Agricultural Chemistry, Faculty of Science University of Alicante Alicante Spain

**Keywords:** betalains, chemical partitioning, cellular antioxidant activity, DNA protection assay, HPLC‐DAD‐MS/MS

## Abstract

The increasing global demand for diverse and health‐promoting foods has led to the expansion of tropical fruit cultivation beyond their native regions, notably into the Mediterranean area. This shift necessitates a deeper understanding of their phytochemical profiles, as environmental factors in new cultivation contexts can significantly influence the biosynthesis of their bioactive compounds. In this study, we explored the phytochemical and antioxidant properties of pitaya fruit, focusing on chemical fractionation and the link between its bioactive components and functional benefits. Using HPLC‐DAD‐MS/MS, key compounds contributing to the fruit's antioxidant effects were identified. The total polyphenol content (TPC) in the original pitaya extract was 670.57 ± 15.98 mg 100 g^−1^ of FW. Both in vitro (ABTS, DPPH, and FRAP) and the cellular antioxidant activity (CAA) assay demonstrated a dose‐dependent prevention of oxidative damage, with 50% inhibition (IC_50_) observed at 2.365 ± 0.072 µg/mL for ABTS, 20.797 ± 0.950 µg/mL for DPPH, and 7.28 ± 0.050 µg/mL for the CAA assay. Additionally, the DNA protection assay revealed that pitaya extract significantly reduced oxidative damage caused by H_2_O_2_ and UV exposure, with a significant reduction in damage at the lowest tested concentration (100 µg/mL). Following chromatographic fractionation via flash chromatography, the fraction enriched in betacyanin (F2), and betaxanthine (F3) exhibited the strongest antioxidant effects on in solution‐based assays. Interestingly, the flavonoid‐rich fractions (F5 and F7), despite showing lower activity in these solution assays, provided nearly complete protection in cell‐based biological models, raising the possibility that additional, and/or complementary antioxidant mechanisms could be involved. These findings underscore the potential of pitaya fruit as a valuable natural source of antioxidants for nutraceutical and therapeutic applications aimed at counteracting oxidative stress and related pathologies.

**Practical Applications:**

1. Betalains, in the F2 fraction, play a key role in the antioxidant potential of pitaya fruit.

2. DPPH and ABTS assays rank pitaya among the fruits with high antioxidant potential.

3. Pitaya fruit extract preserves up to 90% of DNA integrity against oxidative insults.

4. 80% of detected bioactive compounds were betalains and 20% were flavonoids.

## 1 Introduction

Pitaya (*Hylocereus hybridum*, Cactaceae family) is a fruit crop largely grown in tropical and subtropical regions. Originating from Central and South America, pitaya has gained international renown because of its unique appearance and health potential (Suárez‐Cáceres et al. 2024). In recent years, interest in pitaya cultivation has also spread to tropical and subtropical regions, coming into Mediterranean areas, where conditions have become more favorable to its growth (Trindade et al. 2023). Despite this, the tropical fruit remains little known in Europe (Trindade et al. 2023; Suárez‐Cáceres et al. 2024). The ones for the pulp's colorful shades are some bioactive compounds, namely betalains, which not only contribute to the fruit's appealing color, but also have remarkable functional properties (Corimayhua‐Silva et al. 2024; Belmonte‐Herrera et al. 2022). Concerning this aspect, current research on pitaya is a rapidly expanding field, driven by its increasing global popularity as a nutritious and exotic fruit. A primary focus lies in comprehensively elucidating its rich phytochemical composition, with particular attention to betalains, phenolics, flavonoids, and vitamins, which are recognized for their potent antioxidant properties (Paśko et al. 2021). Beyond simple scavenging, studies are increasingly investigating pitaya's diverse bioactivities, including anti‐inflammatory, anti‐diabetic, and potential anti‐cancer effects, aiming to understand the specific compounds responsible for these health‐promoting attributes (Huang et al. 2021). For instance, a recent study showed that consuming pitaya may have potential applications in the prevention of protein glycation associated with diabetes, which plays a significant role in the aging process and age‐related diseases (Ravichandran et al. 2021), cardiovascular risk (Cheok et al. 2022), neuronal damage (Thwe et al. 2023), and chronic inflammation (Nishikito et al. 2023). These beneficial effects are related to the regulation of oxidative stress and the modulation of inflammatory and metabolic cellular processes (Ravichandran et al. 2021). In addition, recent studies have suggested that the bioactive components of pitaya can also modulate the genetic expression of enzymes involved in cellular detoxification mechanisms, such as glutathione peroxidases and catalases, improving the cells' ability to counteract oxidative stress (Nishikito et al. 2023). These mechanisms open up new prospects for the use of pitaya not only as a functional food or foodstuff (Shah et al. 2023), but also as a potential preventive intervention in diseases related to aging and metabolic imbalance.

The objective of this study is to characterize the chemical and functional profile of a variety of pitaya cultivated in the Sicilian territory (var. Costa Rica sunset), with a specific focus on the identification of its main secondary metabolites and the evaluation of its biological properties. The choice of *Hylocereus hybridum* var. Costa Rica sunset cultivated in Sicily is motivated by the increasing spread of this variety in the Mediterranean area, where it has shown good adaptability to local agro‐climatic conditions, characterized by hot summers, low rainfall, and volcanic soils (Trindade et al. 2023). These environmental characteristics directly influence plant physiology and fruit composition, making the study particularly relevant to the European context (Sgroi et al. 2023). In addition, the variety represents an interesting opportunity for the development of innovative and sustainable fruit supply chains in non‐tropical contexts, which are currently still poorly investigated in the literature. The in‐depth study of the characteristics of this cultivar in an emerging growing area can therefore help to define targeted agronomic strategies and enhance local production (Sgroi et al. 2023). In particular, through the use of high‐performance liquid chromatography (HPLC) and mass spectrometry (MS/MS) techniques, we identified and quantified the main bioactive compounds present in the pulp. In addition, by chemical fractionation with flash chromatography, the pulp extract and its constituents were divided into different fractions. At the functional level, we used solution assays to determine the antioxidant mechanism of the compounds identified for each fraction, through which these compounds exert their antioxidant action. In addition to the redox‐active properties, we evaluated the functional properties of Pitaya in biological models to verify the prevention of DNA damage and whole‐cell oxidation (CAA assay).

## 2 Materials and Methods

### 2.1 Plant Materials and Extract Preparation

Pitaya (*H. hybridus)* fruits were collected at Vivai Torre (Milazzo, Sicily, Italy; 38°19′ N, 15°24′ E; 20 m a.s.l.) and identified taxonomically by Giancarlo Torre (botanist). The fruits were frozen at ‐80°C until the extracts were prepared. For the extract preparation, the fruits were thawed, chopped, and homogenized using a mechanical blender (Waring Commercial Blender, CB‐608D, USA). After estimating the moisture content, 70% (v/v) ethanol was added to reach a 1:10 (w/v) ratio. Samples were vortexed for 5 min and sonicated at 4°C for 15 min. After centrifugation (10 min at 8000 g, 4°C), the supernatants were filtered and stored at −20°C. The whole procedure was repeated five times to obtain separate technical replicates.

### 2.2 Phytochemical Characterization via UV/Vis Assays

#### 2.2.1 Total Polyphenol Content (TPC)

TPC was measured by monitoring the color development at 725 nm caused by the reduction of the mixture of tungsten and molybdenum oxides (Folin‐Ciocalteu reagent), as previously described (Mannino et al. 2022). Quantification was performed using gallic acid (GA) as a standard. The results were expressed as mmol of GA equivalents (GAE) per 100 g of fresh weight (FW).

#### 2.2.2 Total Flavonoid Content (TFC)

TFC was measured based on the nitration of aromatic rings with an unsubstituted or sterically encumbered catechol group, as previously described (Shraim et al. 2021). The results were expressed as mmol of rutin equivalent (RE) per 100 g of FW.

#### 2.2.3 Total Proanthocyanidin Content (TPAC)

TPAC was measured by monitoring the formation of the green chromophore resulting from the reaction of proanthocyanidins (PAC) with 4‐dimethylaminocinnamaldehyde (DMAC) reagent (Mannino et al. 2021). Quantification was performed using A2‐type PAC (PAC‐A2) as a standard, and the results are expressed as PAC equivalent (PACE) per 100 g of FW.

#### 2.2.4 Total Anthocyanin Content (TAC)

TAC was measured by monitoring the color variation of anthocyanins as a function of pH, as previously described (Mannino et al. 2019; Campobenedetto et al. 2021). The results were expressed as mg cyanidin‐3‐glucoside equivalent per gram using the extinction coefficient factor (ε = 49,700 L·mol^−1^cm^−1^) and molecular weight (MW = 449.7 g·mol^−1^) of cyanidin glucoside. The dilution ratio (*D*_f_), the light path length (*l*), the extraction volume (*E*_v_), and the amount used for the extract preparation (*g*) were also considered to calculate TAC following Equation 1:

(1) $$\begin{array}{ccc} \mathrm{TAC}\; & = & \frac{{\left(\mathrm{Ab}s_{520}-\;\mathrm{Ab}s_{720}\right)}_{\mathrm{pH}1.0}-{\left(\mathrm{Ab}s_{520}-\;\mathrm{Ab}s_{720}\right)}_{\mathrm{pH}4.5}}{\varepsilon lg}D_{f}\;\mathrm{MW}\;\\ & & E_{v}1000 \end{array}$$

#### 2.2.5 Total Betalain (TBC) and Vulgaxanthin (TVC) Content

TBC and TVC were calculated spectrophotometrically, diluting the sample in a 1:10 ratio with 0.05 M phosphate buffer at pH 6.5 (Campobenedetto et al. 2021). Absorbance at 538 nm and 476 nm were recorded to respectively quantify betanin (Equation 2) and vulgaxanthin (Equation 3). In addition, absorbance at 720 nm was monitored to correct for false positives generated by background noise.

(2) $$\mathrm{TBC}\;=\frac{\left(\mathrm{Ab}s_{540\mathrm{nm}}-\mathrm{Ab}s_{720\mathrm{nm}}\right)}{\varepsilon_{\mathrm{bet}}lg}D_{f}\;MW_{\mathrm{bet}}E_{v}1000$$

(3) $$\mathrm{TVC}\;=\frac{\left(\mathrm{Ab}s_{480\mathrm{nm}}-\;\mathrm{Ab}s_{720\mathrm{nm}}\right)}{\varepsilon_{\mathrm{vulg}}lg}\;\;D_{f}\;MW_{\mathrm{vulg}}E_{v}1000$$

where Abs represent the absorbance at specific wavelengths (480, 540, or 720 nm), MW represents the molecular weight of betacyanin (550.47 g mol^−1^) or vulgaxanthin (339.10 g mol^−1^), while ε stands for the molar extinction coefficient of betacyanin (60,000 L·mol^−1^·cm^−1^), or vulgaxanthin (48,000 L·mol^−1^·cm^−1^). The dilution ratio (D_f_), the light path length (l = 1 cm), the extraction volume (E_v_), and the amount used for the extract preparation (g) were also considered.

### 2.3 Identification and Quantification of Bioactive Compounds via HPLC‐DAD‐MS/MS

Chromatographic analysis was performed using an HPLC instrument from Agilent Technologies (model 1200) coupled with a DAD and a tandem mass spectrometry (MS/MS) system featuring an Agilent 6330 Series Ion Trap LC‐MS. The chromatographic separation utilized a reverse‐phase C18 Luna column (3.00 µm particle size, 150 mm × 3.0 mm internal diameter, Phenomenex) operated at a constant flow rate of 0.2 mL min^−1^ and maintained at a temperature of 25°C within an Agilent 1100 HPLC G1316A Column Compartment. For flavonoid identification and quantification, tandem mass spectrometry analyses were conducted in negative ionization mode, while for betalains, positive ionization mode was employed. Chromatographic conditions and mass spectrometer parameters were optimized for the accurate determination of these compounds, as previously reported (Bajpai et al. 2016).

### 2.4 Evaluation of Antioxidant Properties

#### 2.4.1 Radical‐Scavenging Activity

Radical‐scavenging activity was measured via DPPH (2, 2‐diphenyl‐1‐picrylhydrazyl) (Brand‐Williams et al. 1995) and ABTS (2, 2'‐azino‐bis acid) (Re et al. 1999) assays. Briefly, the decay of 10 mM radical DPPH and 7.5 mM radical ABTS was monitored after the addition of different sample dilutions, with measurements taken at 517 or 734 nm, respectively. For both assays, the inhibition percentage of the color decay (CD%) was assessed using Equation 4, and the obtained curves were plotted as a function of concentration to determine the sample amount required to achieve 50% color inhibition (IC_50_) by linear regression. Results were expressed as mmol of Trolox equivalents (TE) by comparing the sample data with that obtained from dose‐response curves using 6‐hydroxy‐2,5,7,8‐tetramethylchroman‐2‐carboxylic acid (Trolox) as a standard.

(4) $$\mathrm{CD}\%\;=\frac{\left(\mathrm{Ab}s_{\mathrm{blank}}-\;\mathrm{Ab}s_{\mathrm{sample}}\right)}{\mathrm{Ab}s_{\mathrm{blank}}}100$$

#### 2.4.2 Metal‐Reducing Antioxidant Power

The reducing antioxidant power was evaluated via ferric‐reducing antioxidant power (FRAP) assay (Benzie and Strain 1996), monitoring the ability of the compounds present to reduce ferric ions (Fe^3+^) to ferrous ions (Fe^2+^), forming a complex detectable spectrophotometrically at 593 nm. Results were expressed as mmol of Trolox equivalents (TE) per 100 g of FW, using an external calibration curve.

#### 2.4.3 DNA Protection Assay

A DNA protection assay was performed evaluating the ability of samples to protect plasmid DNA (pUC19) breakage by hydroxyl radicals under UV exposure, as previously described (Tsai et al., 2019). Briefly, after transforming *Escherichia coli* via the heat shock method with plasmid pUC19, it was cultured in capsules containing 50 mL of 1% (w/v) agarose gel supplemented with 1% (v/v) kanamycin for 24 h at 37°C in dark conditions. After incubation, a single colony was selected and transferred into tubes containing liquid growth medium supplemented with 1% (v/v) kanamycin. After 24 h incubation at 37°C, bacterial cells were collected, and pure plasmid DNA was extracted using the PureLink Microbiome DNA Purification Kit (Thermofisher, Milan, Italy) according to the manufacturer's instructions. Purified pUC19 plasmid was incubated with 50 mM H_2_O_2_ and various dilutions of sample extracts, then exposed to direct UV‐light for 15 min. After incubation, 6X DNA loading buffer (Thermofisher, Milan, Italy) was added to each microtube, and the samples were loaded into 1% (w/v) agarose gel pre‐stained with Gel Red (Thermofisher, Milan, Italy). Electrophoresis was conducted at 90 V for 30 min, and DNA bands were visualized using a Benchmark SmartBlue Mini Transilluminator (VWR International, Milan, Italy).

#### 2.4.4 Cellular Antioxidant Activity (CAA)

The CAA assay was performed using the HepG2 cell line (American Type Culture Collection ATCC, Rockville, MD, USA) cultured in RPMI medium supplemented with 5% (w/v) FBS, 2 mM L‐glutamine, penicillin (50 IU/mL) and streptomycin (50 µg/mL). During growth conditions in 75 cm^2^ flasks, cells were maintained in a humidified atmosphere at 5% CO_2_ at 37°C. In order to perform the CAA assay, the cells were trypsinized and seeded at a density of 6.0 × 10^4^ cells/well in 96‐well plates, as previously described (Campobenedetto et al. 2020). After 24 h, 25 µM of 2, 7‐dichlorofluorescin diacetate (DCFH‐DA) and various concentrations of extracts were added. After 2 h, they were washed and incubated with 600 µM of 2, 2‐azobis (2‐amidinopopane) dihydrochloride (ABAP) in Hank's Balanced Salt Solution (HBSS). The antioxidant activity was evaluated by the fluorescence emission resulting from fluorescent 2’, 7’‐dichlorofluorescein (DCF) every 5 min for 1 hr. CAA value was calculated by integrating the area under the curve of fluorescence obtained for samples and normalized for blanks (∫SA) and the integrated area under the curve of fluorescence obtained for controls and normalized for blank (∫CA) using Equation 5. Finally, the concentration necessary to inhibit 50% of 2’, 7’‐dichlorofluorescin (DCF) formation (CAA50) for each fruit extract was calculated from concentration/response curves using linear regression analysis. Data were expressed as CAA_50_ (mg of FW per mL cell medium). The experiments were repeated three times.

(5) $$\mathrm{CAA}=100-\frac{\int \mathrm{SA}\;}{\int \mathrm{CA}}100$$

### 2.5 Chemical Fractionation via Flash Chromatography

The same extracts used for the phytochemical characterization and the evaluation of functional properties were concentrated at a controlled temperature using a CentriVap system to ensure the complete removal of the organic solvent while preventing degradation of thermolabile compounds. Subsequently, residual water was removed by freeze‐drying, resulting in a powdered sample. The resulting powder was then resuspended in 90% (v/v) methanol, and the mixture was sonicated on ice for several minutes to promote complete dissolution. A 50 mL aliquot of this solution was loaded onto the top of a Biotage Sfär C18 D (300 g, 150 mm height, 50 mm i.d., 30–50 µm particle size) mounted on a Flash Chromatography System (Biotage Selekt System). Chromatographic separation was performed using 0.5% (v/v) formic acid (Solvent A) and acetonitrile acidified with 0.5% (v/v) formic acid (Solvent B). The flow rate was set at 40 mL/min, and the gradient was optimized to maintain Solvent B at 5% (v/v) for the initial 5 min and then raised from 5% (v/v) to 90% (v/v) in a 35‐minute period. During separation, 50 fractions were collected with a time interval of 0.5 min for each fraction, ensuring accurate fractionation of the compounds in the extract. Chromatographic spectra were monitored in the UV/Vis range of 200 to 720 nm, allowing detection of absorbing compounds in that wavelength range. Each fraction was then analyzed for total phenolic content using the Folin–Ciocalteu method, and UV/Vis absorbance profiles were evaluated to assess similarities in spectral features (e.g., λmax and absorbance shape). Fractions showing comparable retention times, similar UV/Vis spectra, and overlapping antioxidant activity were grouped, resulting in eight pooled fractions (F1–F8). Grouping was also guided by elution polarity, estimated based on the solvent composition during fraction collection and expressed as polarity index values. The polarity index ranged from approximately 9.8 for the earliest fraction (F1), indicating highly polar conditions, gradually decreasing through intermediate fractions (F2: 9.2; F3: 8.6; F4: 8.0; F5: 7.4) to less polar conditions in the later fractions (F6: 6.8; F7: 6.3; F8: 6.2). Accordingly, earlier fractions (F1–F3) contained more polar compounds, while later fractions (F6–F8) were enriched in less polar, likely lipophilic constituents. A representative chromatogram and the grouping rationale are included in Figure 3 Panel A.

### 2.6 Statistical Analysis

All data are presented as the mean ± standard deviation (SD), calculated from five independent replicates. The Student t‐test or one‐way ANOVA, followed by Tuckey's post hoc test, were performed to assess significance among the different experimental conditions. A value of *p* < 0.05 was predetermined as the criterion of significance. All statistical analyses were carried out using SPSS Statistics 24 (SPSS, Chicago, IL, USA).

## 3 Results and Discussion

### 3.1 Phytochemical Characterization of Pitaya Fruit Extract via UV/Vis Assay

**FIGURE 1:**
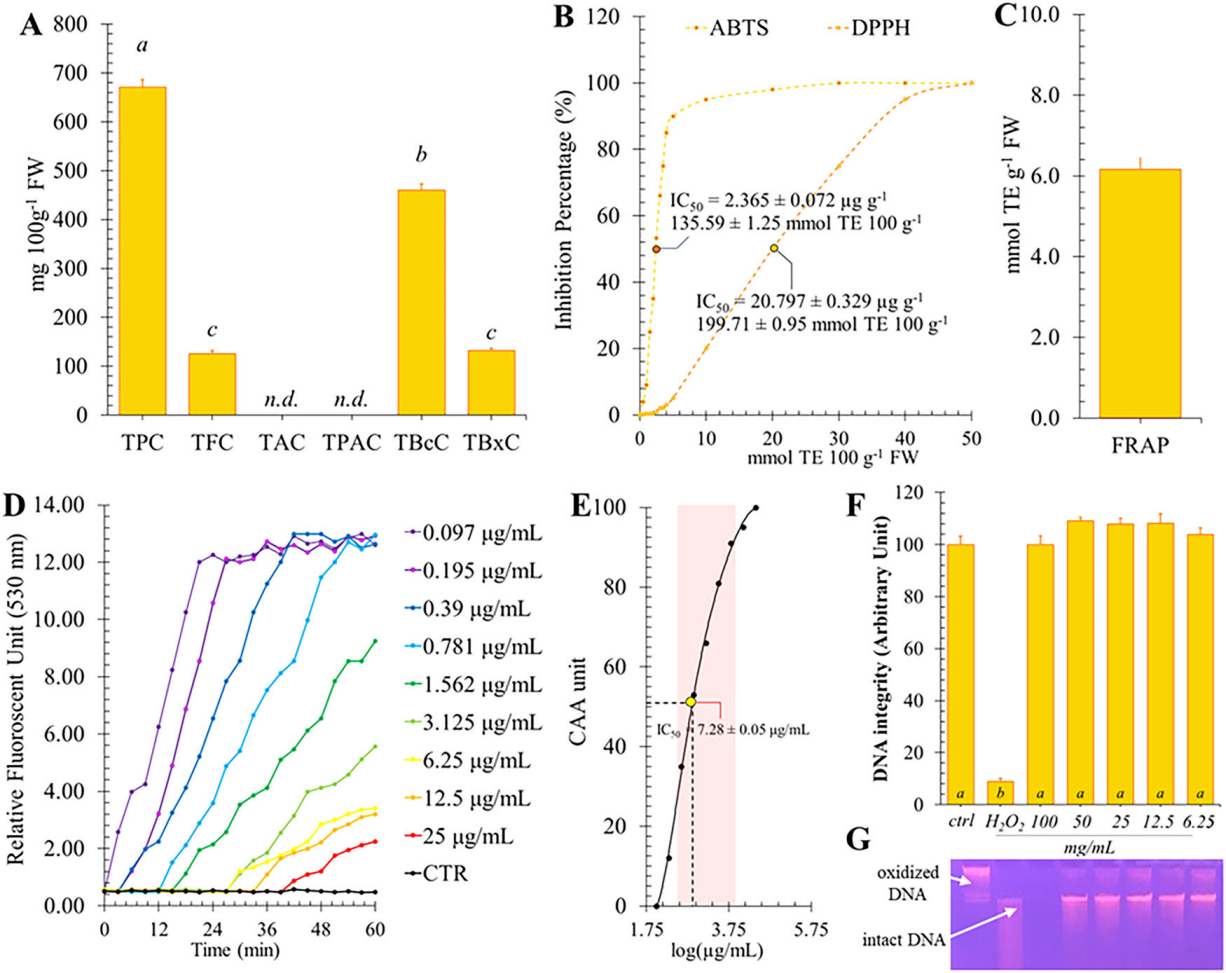
UV/Vis phytochemical characterization and evaluation of antioxidant potential of pitaya pulp extract. Panel A shows the total content of polyphenols (TPC), total flavonoids (TFC), anthocyanins (TAC), proanthocyanidins (TPAC), betacyanins (TBcC), and betaxanthin (TBxC) expressed as mg per 100 g of FW. Panel B shows radical scavenging activity evaluated by ABTS and DPPH assay. For this panel, data are expressed both as IC_50_ (µg g^−1^) and mmol TE 100g^−1^. Panel C shows the reducing activity evaluated by FRAP assay, expressed as mmol TE g^−1^. Panel D shows the raw kinetic data from the cellular antioxidant activity (CAA) assay, which were used to generate the dose‐response curve depicted in Panel E. From this, the CAA_50_ value was determined as µg mL^−1^ of cell medium. Panel F displays the results of the DNA oxidative damage assay, showing the effects of H_2_O_2_ alone on DNA integrity and the impact of co‐incubation with different concentrations of pitaya fruit extract. Panel G presents the DNA electrophoresis gel used to generate the quantitative data shown in Panel F.

**FIGURE 2:**
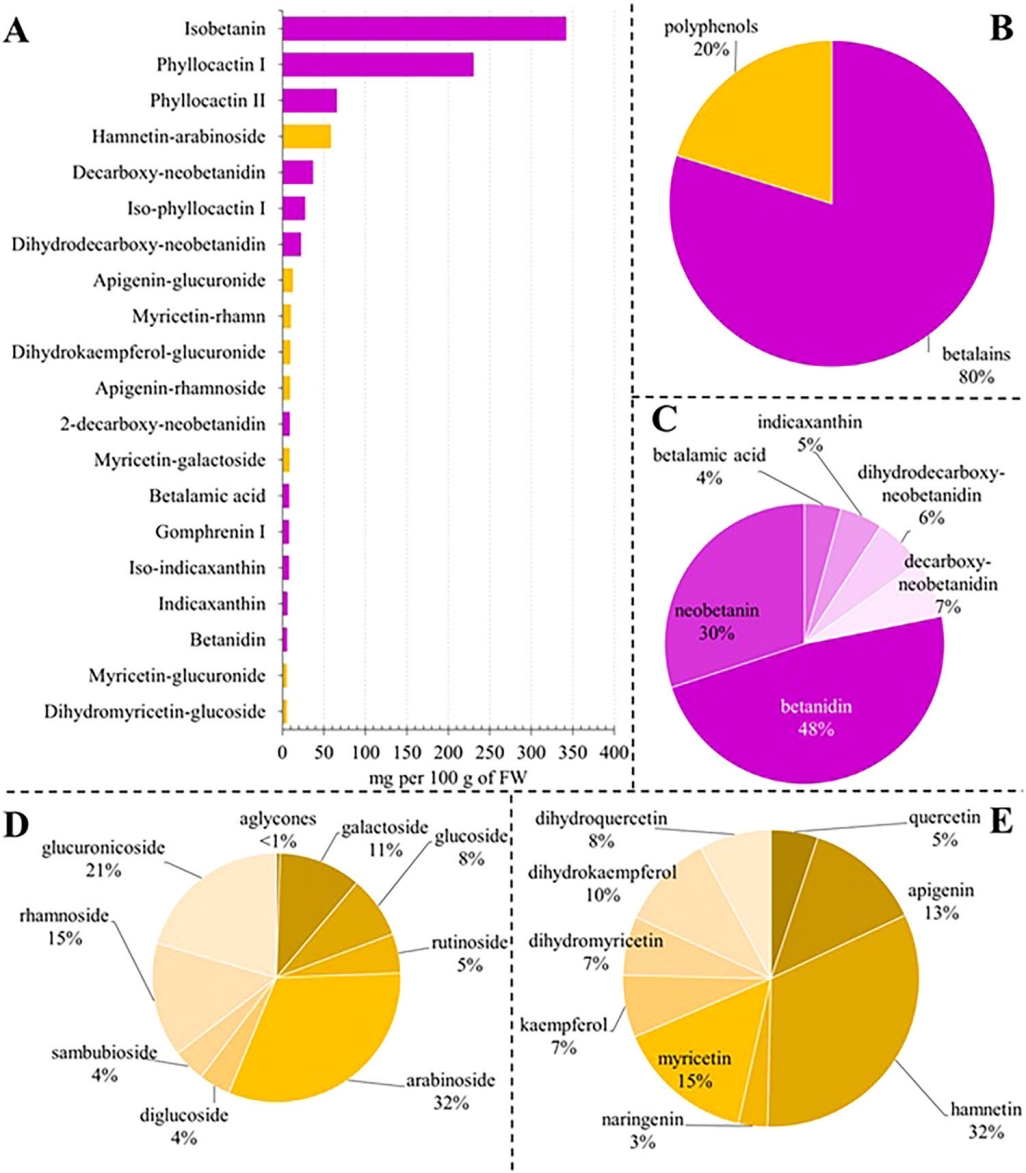
Phytochemical profiling of pitaya fruit extracts by HPLC‐DAD‐MS/MS. Panel A shows the top 20 compounds identified and quantified in pitaya fruit extract. Data are plotted as mg per 100 g of FW. Panel B illustrates the percentage distribution between polyphenols and betalains, while in Panel C the percentage distribution of the different betalain compounds is depicted. Panel D analyzes the polyphenolic compounds according to the degree of glycosylation, while Panel E presents the percentage distribution according to the flavonoid scaffold.

This study investigated the phytochemical profile of pitaya fruits using both UV/Vis and HPLC‐DAD‐MS/MS analysis. The results of spectrophotometric determinations are shown in Figure 1 along with the evaluation of antioxidant potential, while identification and quantification of bioactive compounds are reported in Figure 2. Interestingly, the TPC value measured in pitaya pulp extracts (Figure 1A) exceeded that reported for most of the fruits included in the Phenol‐Explorer Database, ranking among the top 30 fruits with the most valuable content (670.57 ± 15.98 mg 100 g^−1^ of FW) (Pérez‐Jiménez et al. 2020). However, it is well established in the literature that the TPC value is not exclusively linked to the presence of polyphenolic compounds, but rather provides a broader indication of the overall antioxidant capacity of a fruit extract. Indeed, the reaction on which the TPC is measured via the Folin‐Ciolteau assay involves the detection of Tg and Mb in their reduced state. Therefore, the TPC value can be affected not only by polyphenols, but also by any other compound having a non‐polyphenol scaffold with antioxidant capacity, either directly or indirectly (Mantaenu and Apetrei 2021). This makes TPC useful for comparisons between different extracts, but not specific to polyphenols alone. More specialized assays based on chemical reactions have been developed to more accurately determine the presence of specific bioactive compounds and quantify their overall content (Sasidharan et al. 2011). These include the ferric chloride assay for flavonoids, and the pH differential method for anthocyanins, betalains, and betaxanthins, while the DMAC method for proanthocyanidins. Each of these assays provides useful information on the qualitative and quantitative profile of bioactive compounds, offering a more precise indication to guide future targeted chromatographic analyses.

Within polyphenols, flavonoids stand out as particularly noteworthy due to their widespread occurrence and well‐documented properties (Chen and Chen 2013). These compounds, characterized by a basic structure consisting of two aromatic rings bound together by three carbon atoms, not only contribute to the appealing colors of flowers, fruits, and leaves but also exhibit significant antioxidant activity, which helps combat oxidative stress and reduce the risk of chronic diseases (Mohammed et al. 2014). This group of metabolites has been found in pulp with a concentration of 125.31 ± 6.37 mg per 100 g of FW (Figure 1A). However, as suggested by TAC and TPAC (Figure 1A), anthocyanins and proanthocyanidins were not detected in the samples. Anthocyanins and proanthocyanidins are two groups of flavonoid compounds of great interest for their antioxidant properties and for their role in imparting color to flowers and fruits (Pandey and Rizvi 2009). Anthocyanins are water‐soluble pigments responsible for blue, purple, and red hues in plants and are derived from the modification of a basic structure called anthocyanidin. On the other hand, proanthocyanidins are oligomers of flavan‐3‐ols playing a key role in color stability and plant protection against pathogens (Rauf et al. 2019). Both groups of compounds share a common biosynthetic pathway, known as the flavonoid pathway, which starts from the precursor compound cinnamic acid and goes through various stages of enzymatic modification to produce anthocyanins and proanthocyanidins (Liu et al. 2021). Although numerous studies suggest that anthocyanins and proanthocyanidins can co‐exist in certain plant species that also produce betalains (Sakuta et al. 2021), this idea deserves reconsideration. The physiological pathways in plants typically promote a mutual self‐exclusion between these two classes of bioactive molecules, making their simultaneous synthesis unlikely. Indeed, betalains and anthocyanins share the same molecular precursors, and when betalains are synthesized, their production can inhibit the activity of enzymes required for the production of anthocyanins and proanthocyanidins (Sakuta et al. 2021).

Concerning betalains, our analysis indicated a strong presence of betalains, recording a content of 295.93 ± 17.29 mg per 100 g of FW (Figure 1A). Betalains are natural pigments that include betacyanins (red‐purple) and betaxanthins (yellow). Our determinations revealed that 65% of this content was betacyanin‐related compounds, while only 35% was betaxanthins. Betacyanins and betaxanthines represent distinct classes of pigments found in various plant species, both originating from a common biochemical pathway. Due to their chemical scaffold consisting of the condensation of betalamic acid with cyclo‐DOPA (for betacyanins) or the amino acids (betaxanthin), these compounds exert a variety of beneficial properties, including antiproliferative, antimicrobial, and antioxidant properties (Stintzing et al. 2005). Some other tropical fruits, such as prickly pear and xoconostle, also show high levels of betalains. Prickly pear, which generally records an average content of betalain between 16.5 and 214 mg per 100 g of FW, is widely cultivated for its nutritional and medicinal properties (Stintzing et al. 2005; Calva‐Estrada et al. 2022), while the more acidic fruit xoconostle (between 3.71 and 52.31 mg per 100 g of FW) is used in traditional Mexican cuisine and medicine (Morales et al. 2015; Lopez Martinez et al. 2015). In these fruits, betalains influence not only visual appearance but also their potential as functional foods due to their antioxidant and antimicrobial properties (Morales et a. 2015).

### 3.2 Phytochemical Characterization of Pitaya Fruit Extract via HPLC‐DAD‐MS/MS Analysis

Although pitaya is a renowned source of betalains, as suggested by spectrophotometric assays (Figure 1A), other bioactive compounds may also be present. Accordingly, HPLC‐DAD‐ESI‐MS/MS was used to separate and determine the individual bioactive compounds present in pitaya extract. Chromatographic analysis revealed the presence of 48 different compounds, the identification and relative quantification of which are shown in Table S1. Instead, Figure 2A shows the top 20 compounds with the highest content detected within the pitaya extract. Interestingly, about 80% of the detected amount belonged to the betalain class, while the remaining 20% belonged to flavonoids (Figure 2B). Most betalains in red pitaya are derived from betanidin and neobetanin (Figure 2C). Minor pigments include indicaxanthin, accounting for only 7% (w/w) by weight. Indicaxanthin is a yellow pigment with antioxidant and antiradical properties, which have been demonstrated in various in vitro and in vivo studies. This pigment can be found in a few edible plants, such as cacti and some berries. Decarboxy‐neobetanidine and dihydro‐decarboxy‐neobetanidine compounds account for only 5–6% by weight, which emerge as natural degradation products formed through oxidative processes (Wybraniec et al. 2009). Other decarboxylated forms of betanin, such as decarboxy‐neobetanidine, may offer unique bioactivities despite their low concentration (Polturak and Aharoni 2018). Prominent among the betalains is isobetanine, an isomer of betanine known for its cytotoxic potential, which has been studied for possible therapeutic applications, especially in oncology (Miraj 2016; Vieira Teixeira da Silva et al. 2019; Madadi et al. 2020). Other significant compounds are Phyllocactin I and Phyllocactin II, pigments that contribute to fruit coloration and possess bioactive properties, including antimicrobial, antiproliferative, and anti‐viral (Wijesinghe and Choo 2022). Comparing spectrophotometric and HPLC‐MS/MS data for betalains highlights their distinct analytical principles: spectrophotometry assesses total chromophores, which other compounds can influence, while HPLC‐MS/MS specifically identifies and quantifies individual betalains.

As for polyphenols, most of the compounds in pitaya extracts are bound to sugar moieties (Figure 2D). The biological activity of phenolic compounds can be strongly influenced by their structure and glycosylation pattern. The aglycone is often associated with therapeutic effects, while the glycosidic moiety enhances water solubility and pharmacokinetic and pharmacodynamic properties (Chuang et al. 2017). In pitaya fruit extracts, the phenolics arabinosides account for 32% of the total, followed by glucuronicosides (21%), rhamnosides (15%), and galactosides (11%). Other glycosides, including glucosides, rutinosides, sambubiosides, and diglucosides, together account for less than 20%. In addition, aglycones make up less than 1% of the extract (Figure 2D). In addition to the degree of glycosylation, chemical changes to the structure also influence the bioactivity of flavonoids. Among the major phenolic compounds detected in pitaya extracts, rhamnetin, myricetin, and apigenin stand out (Figure 2E). Among these, rhamnetin‐arabinoside is identified as the fourth most abundant compound in pitaya extract and the most abundant polyphenol (Figure 2E). Rhamnetin is a flavonoid belonging to the class of O‐methylated flavonols, and is known for its antioxidant and anti‐inflammatory properties (Lee et al. 2022). When bound to arabinose, the resulting glycoside improves the compounds’ solubility and bioavailability (Schaub et al. 2021). The second most abundant flavonoid was apigenin‐glucuronide, a flavone widely studied for its pharmacological properties, including anticancer, antioxidant, and anti‐inflammatory activities (Ali et al. 2017). This compound is present in similar concentrations in several common fruits and vegetables such as grapefruit, parsley, onions, and corn (Ali et al. 2017; Salehi et al. 2019). Myricetin, found in its rhamnoside and galactoside forms, ranks as the 9th and 12th most abundant compounds, respectively (Figure 2E). Myricetin is a flavonol with recognized antioxidant and anti‐inflammatory properties, and is commonly found in berries, garlic, and fruits such as currants and guava. Its chemical structure, characterized by two aromatic rings connected by a chain of three carbon atoms, contributes to its effectiveness in neutralizing free radicals (Agraharam et al. 2022).

### 3.3 Antioxidant Potential of Pitaya Fruits Extract

Preliminary spectrophotometric characterization carried out on pitaya extracts suggests that this fruit possesses other antioxidant macromoles in addition to the classical antioxidant vitamins, ranging from N‐containing compounds to polyphenols. Furthermore, considering that the concept of total antioxidant capacity of foods accounts for additive, synergistic, and/or antagonistic redox interactions among the different molecules present, it is very important to employ spectrometric determinations to get an overall idea of their potential (Sonter et al. 2021; Yu et al. 2021; dos Santos Souza et al. 2024). Whereas it has been reported that a single assay is not sufficient to predict the antioxidant potential of plant extracts, the results of several assays can help to elucidate the mechanisms involved in the observed activities (Munteanu and Apetrei 2021). Consequently, in order to assess the overall intrinsic antioxidant capacity of pitaya fruit extracts, we employed three different antioxidants in solution assays, namely ABTS, DPPH (Figure 1, Panel B), and FRAP (Figure 1, Panel C) assays. Despite criticism due to the evident limitations of in vitro chemical methods for determination of antioxidant properties of fruit extracts, these assays are very popular for technological and nutritional purposes since they provide valuable information on the complex mixture of redox active molecules. In this context, our results showed that pitaya extracts possess high radical scavenging properties. In particular, the value obtained by the DPPH assay was higher than that obtained by ABTS. The observed differences could be explained by the variability in pH or hydrophilicity of the reaction mixtures, and the relative difference in the scavenging ability of the antioxidant compounds present in the extracts (Cano et al. 2023). In addition, although lower than grape red, plum black, and strawberry, these values were much higher than watermelon, pear, nectarine, mango, and avocado, and comparable to those reported in popular antioxidant‐rich foods, such as sweet cherry, orange, and lemon (Floegel et al. 2011). Concerning the reducing activity, pitaya recorded higher values than most common fruits for human consumption, and lower only than thornapple (Guo et al. 2003). Although DPPH, ABTS, and FRAP assays are based on the principle of radical scavenging capacity or reducing activity, they are not directly comparable and do not account for the potential bioavailability and efficacy of antioxidants within the cellular systems. Consequently, to achieve a more accurate assessment of the antioxidant activity of dietary compounds in biological contexts, we performed a CAA assay in order to both assess the ability of antioxidants to penetrate cells, counteract oxidation in cellular environments, and reflect more realistic interactions with radical species (Kellett et al. 2018). Indeed, the CAA assay uses HepG2 cells, which, through the expression of phase I and II enzymes, effectively simulate human liver metabolism, allowing realistic assessment of intracellular antioxidant activity. This model allows measurement of effective uptake, metabolism, and protective capacity against oxidative stress within a complex cellular context, ensuring reproducible and physiologically relevant results compared with other cell lines (Kellett et al. 2018; Wolfe and Liu 2007). Under our experimental conditions, when cells were treated with DCFH‐DA and ABAP, oxidation resulted in deacetylation of DCFH‐DA and increased fluorescence (Figure 1D). However, in the presence of antioxidants from red pitaya pulp extract, a dose‐dependent effect can be observed. Specifically, also the lowest concentrations of extract (between 0.097 and 1.397 µg mL^−1^) displayed a reduction in fluorescence that was observed for up to 12 min. In contrast, higher concentrations inhibited oxidation more markedly and for a longer duration (about 40 min), reaching peak fluorescence only after 60 min, with a maximum level of 2.2 (Figure 1D). Based on the subtending areas obtained from the curves shown in Figure 1D, the CAA_50_ of each dosage was calculated, and the total CAA_50_ was determined to be 7.28 ± 0.05 µg mL^−1^ by logarithmic regression (Figure 1E). Compared with the values determined by Wolfe and Liu for several common fruits, the antioxidant capacity measured in our extracts was much higher. In particular, comparing our results with the CAA_50_ value of colored fruits, including several berries, cherries, and pomegranates, pitaya pulp extracts showed 3‐ to 30‐fold higher antioxidant activity. Oxidative stress poses a threat not only to cellular integrity but also to genomic material, as shown by studies on damage caused by light and UV radiation, which can induce the formation of pyrimidine dimers and oxidized bases such as 8‐hydroxy‐2'‐deoxyguanosine (8‐OHdG) (Cortat et al. 2013; Di Minno et al. 2016). Consequently, here we investigated the potential protective effects of pitaya fruit extracts in mitigating oxidative DNA damage and contributing to the development of strategies to combat diseases associated with this type of stress. In order to assess the protective properties of pitaya pulp extract, we exposed plasmid DNA derived from *Escherichia coli* to increasing concentrations of the extract, simulating natural exposure to these compounds. Following this, the DNA samples were subjected to oxidative stress conditions induced by hydrogen peroxide (H_2_O_2_) and direct UV light exposure (Valverde et al. 2018). The plasmid DNA protection assay used is a well‐established method that allows tight control of oxidative stress conditions and provides a uniform DNA substrate, eliminating confounding factors such as cellular metabolism and DNA repair processes. This enables a clear and reproducible assessment of the antioxidant protective capacity of the extracts in vitro (Andonova et al. 2023). By analyzing the integrity of DNA samples after exposure to oxidative stressors, it was observed that all concentrations used had demonstrated a protective effect on DNA integrity (Figure 1F,G). Notably, the samples treated with pitaya pulp extract showed less fragmentation and retained a higher percentage of intact DNA than the control groups. This suggests that the antioxidant properties of the extract effectively mitigated the damaging effects of oxidative stress. In addition, the analysis indicated that all concentrations tested provided some level of protection, including the lowest concentrations. These results underscore the importance of pitaya pulp extract as a natural agent for preserving DNA integrity under oxidative conditions and warrant further investigation into its mechanisms of action and potential applications in health and nutrition. The plasmid DNA protection assay effectively assesses direct oxidation and protective effects on genomic material by bioactive moelcules, but it has biological limitations. This assay focuses mainly on the direct impact and protection of DNA, without fully taking into account other crucial aspects of the cellular response to damage. To gain a more complete understanding of this protective mechanism, future research should consider using a wider range of assays. For example, the COMET assay, also known as single cell gel electrophoresis, could be used to assess DNA damage at the single cell level, offering insights beyond direct oxidation. This broader approach would also allow exploration of DNA damage response and repair pathways and various cellular self‐repair processes (Collins et al. 2023).

### 3.4 Chemical Portioning and Phytochemical Profile of Pitaya Fruit Fractions

**FIGURE 3:**
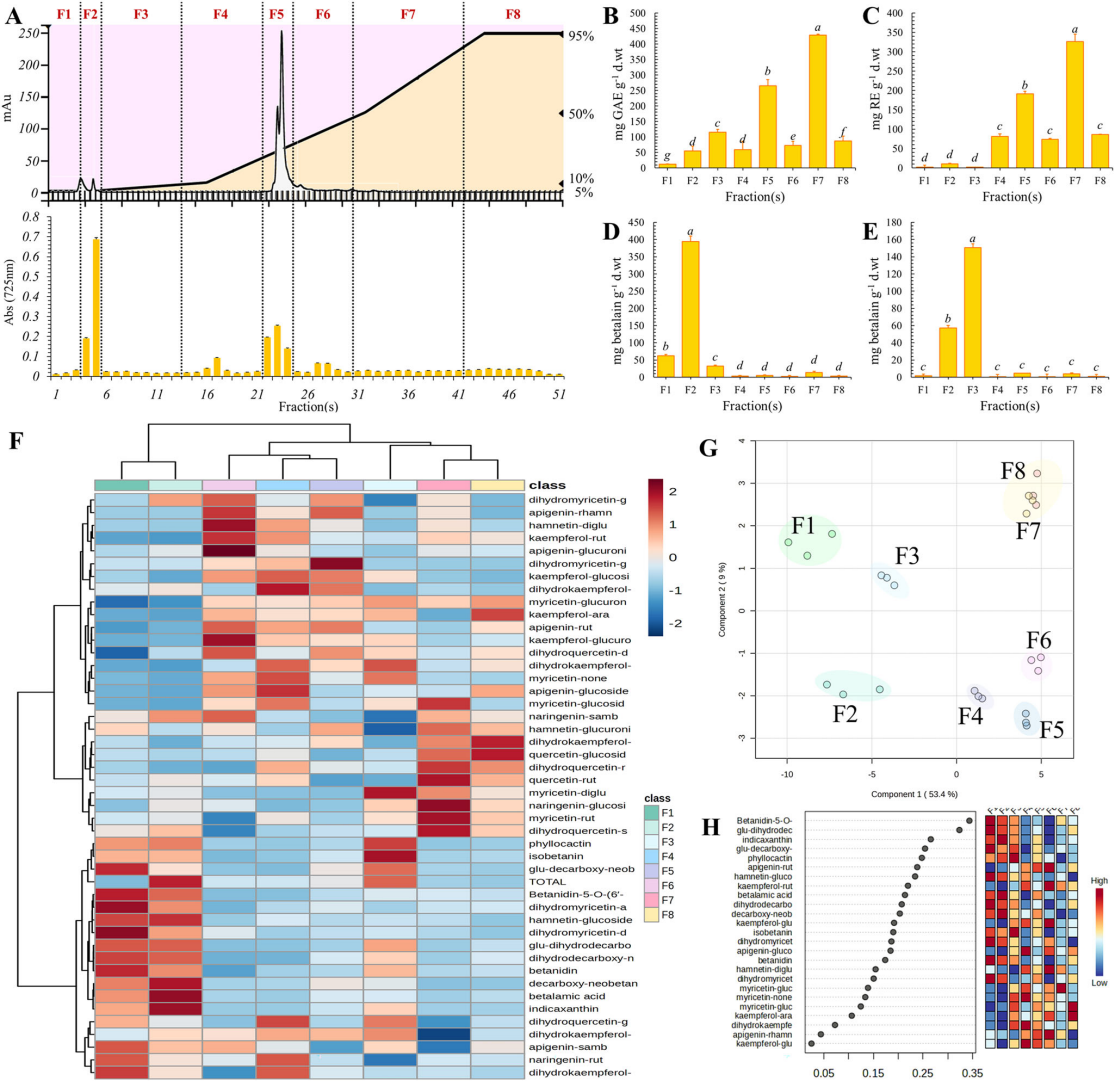
Chemical portioning, UV/Vis Assay and, HPLC‐DAD‐MS/MS analysis of pitaya fractions. Panel A illustrates the fractionation process using Flash Chromatography coupled with Folin‐Ciocalteu analysis to obtain various fractions (F1‐F8). Panels B‐E display the results for total polyphenol content (TPC), flavonoid content (TFC), betacyanin content (TBcC), and betaxanthin content (TBxC) across the different fractions. Panel F presents a heatmap coupled with cluster analysis for the chemical characterization performed via HPLC‐DAD‐MS/MS. Panels G shows the distribution through Principal Component Analysis (PCA), while the key compounds influencing this distribution are reported in Panel H.

Fractionation of bioactive compounds from plant extracts is a fundamental endeavor in phytochemical research, essential for discerning the intricate chemical composition and pharmacological potential of botanical resources (Alfaro Jiménez et al. 2022). In this study, the separation of pitaya pulp extract was performed using Flash Chromatography (Biotage Selektä, Milan, Italy) equipped with a C18 column. During the chromatographic run, continuous monitoring was carried out across the 200–900 nm wavelength range to track the elution of bioactive compounds, including polyphenols and betacyanins. A total of 51 fractions were collected, and the Folin‐Ciocalteu assay was applied to integrate chromatographic data with spectrophotometric analysis (Figure 3A).

In order to evaluate the chemical composition of the fractions of pitaya pulp extract, UV/Vis analysis was employed, showing the distribution of polyphenols, flavonoids, and betalains across the different fractions (Figure 3B–E). Fractions F7 and F5 showed the highest content of polyphenols (429.13 ± 8.32 and 264.85 ± 20.96 mg g^−1^ GAE), followed by F3 (115.47 ± 9.23 g^−1^ GAE) and F2 and F4 (about 50 mg g^−1^ GAE) (Figure 3B). Analysis with the aluminum chloride assay, targeting flavonoids, confirmed F7 and F5 as flavonoid‐rich fractions (325.13 ± 19.21 and 195.77 ± 6.20 mg g^−1^ RE). F2 and F3, despite a moderate polyphenol content according to the Folin‐Ciocalteu assay, showed lower flavonoid levels, suggesting that the detected antioxidant activity might come from non‐polyphenolic compounds. Finally, fraction F1, with low values of TPC, also displayed low TFC (Figure 3C). In addition, higher values of TFC were exclusively observed in the low‐polarity fractions (F5, F6, F7 and F8), which were obtained with an acetonitrile gradient above 30% (Figure 3A–C). Specific analyses for TBcC and TBxC revealed a predominance of betalains in F3 (over 150 mg g^−1^), while F2 contained lower amounts (just over 50 mg g^−1^) (Figure 3D). In contrast, F2 was rich in betaxanthins (3940.55 ± 12.98 mg g^−1^), with lower concentrations in F3 (62.29 ± 4.19 mg g^−1^) (Figure 3D). Except for F1, which showed a modest presence of betaxanthins, the other fractions had TBcC and TBxC contents accounting together for less than 20 mg g^−1^ (Figure 3C–D). In order to investigate the most predominant compounds defining the chemical profile of the different fractions, HPLC coupled with DAD and tandem mass spectrometry was used, as previously applied for the characterization of the extract derived from pitaya fruit (Figure 2). In this case, with the aim to better compare the chemical profile of the different fractions, the quantitative data were normalized to the effective weight of each obtained lyophilized fraction, and then each value was further normalized by the median. The values were then transformed by applying square root, and the data were scaled with centering on the mean and divided by the square root of the standard deviation of each variable (Pareto scaling). The resulting dataset was used to generate a HeatMap coupled with a cluster analysis (Figure 3F) and a PCA distribution (Figure 3G). Moreover, the main bioactive compounds responsible for the different distribution of fractions in the cluster and PCA are shown in Figure 3H.

Cluster analysis and PCA distribution showed the formation of four main groups, as shown in Figure 3G. The first group includes fractions F1 and F3, which are distinguished by positive PCA score‐2 and negative PCA score‐1 values. These two fractions are characterized by having the lowest overall concentration of bioactive compounds, including polyphenols, betalains, or betaxanthins. This observation is in line with previous results obtained by spectrophotometry (Figure 3B–E). In particular, F3 is distinguished by the predominant presence of compounds such as isobetanin, phyllotactin, and several glucosidic forms, indicative of betalaine derivatives or degradation products related to intracellular oxidative processes. Contrastingly, the F2 fraction forms a separate group, characterized by negative values of both PCA score‐2 and PCA score‐1. F2 is distinguished by higher concentrations of indicaxanthin and betalamic acid, along with significant levels of decarboxy‐neobetadinine, hamnetin‐glucoside, and dihydro‐myricetin‐arabinoside (Figure 3H). On the other hand, fractions F4–F8 are separated by positive PCA score‐1 values. Among them, F4–F6 are further defined by negative PCA score‐2, while F7 and F8 form a fourth separate group with positive PCA score‐1 (Figure 3G). Fraction F4, subsequent to F1 in quantitative content, has one of the lowest absolute concentrations of bioactive compounds. The HPLC results showed that the main flavonoids present in appreciable concentrations in F4 are myricetin in aglycone form, kaempferol‐glucoside, and dihydrokaempferol aglycone (Chunhakant, and Chaicharoenpong 2021). In addition, only trace amounts of betalains, predominant in F3, were detected (Figure 3H). The intermediate position of F4 between F3 and F5 suggests that this fraction contains compounds characteristic of both earlier and later fractions, which may not have been effectively separated during chromatography. Within this group, F5 and F6 showed a predominance of polyphenolic compounds. However, chromatographic separation, based on polarity gradient and conformation, showed significant variations in distribution. For example, F5 and F6 showed a strong presence of monoglycerides, while F7 and F8 are found to be enriched in flavonoid aglycones, eluted later due to their lower polarity. The main difference between F7 and F8 lies in the fact that F7 also contains significant amounts of myricetin‐glucoside, myricetin‐riboside, dihydroquercetin‐sambubioside, quercetin‐riboside, naringenin‐glucoside, kaempferol‐rutinoside, myricetin‐glucoside, and dihydro‐myricetin‐diglucoside, which are the most prominent metabolites of these macrofractions (Figure 3F).

### 3.5 Evaluation of Antioxidant Properties of Pitaya Fruit Fractions

In order to have a thorough understanding of the antioxidant activity and its interaction with the phytochemical constituents detected by UV/vis and HPLC analysis in the macro‐fractions (F1‐F8), we performed all experiments on the evaluation of active redox properties using both in solution (ABTS, DPPH, and FRAP) and biological models (CAA and DNAox). By integrating these assessments with phytochemical determinations, it was possible to highlight how the distribution of phytochemicals in pitaya fractions affected antioxidant properties, suggesting a direct correlation between the identified compounds and the antioxidant activities observed in the assays.

Specifically, the results obtained from the evaluation of the antioxidant activity of the different pitaya fractions (F1‐F8) reveal significant differences in their ability to counteract oxidative stress, in relation to the chemical composition and concentration of the bioactive compounds present.

**FIGURE 4:**
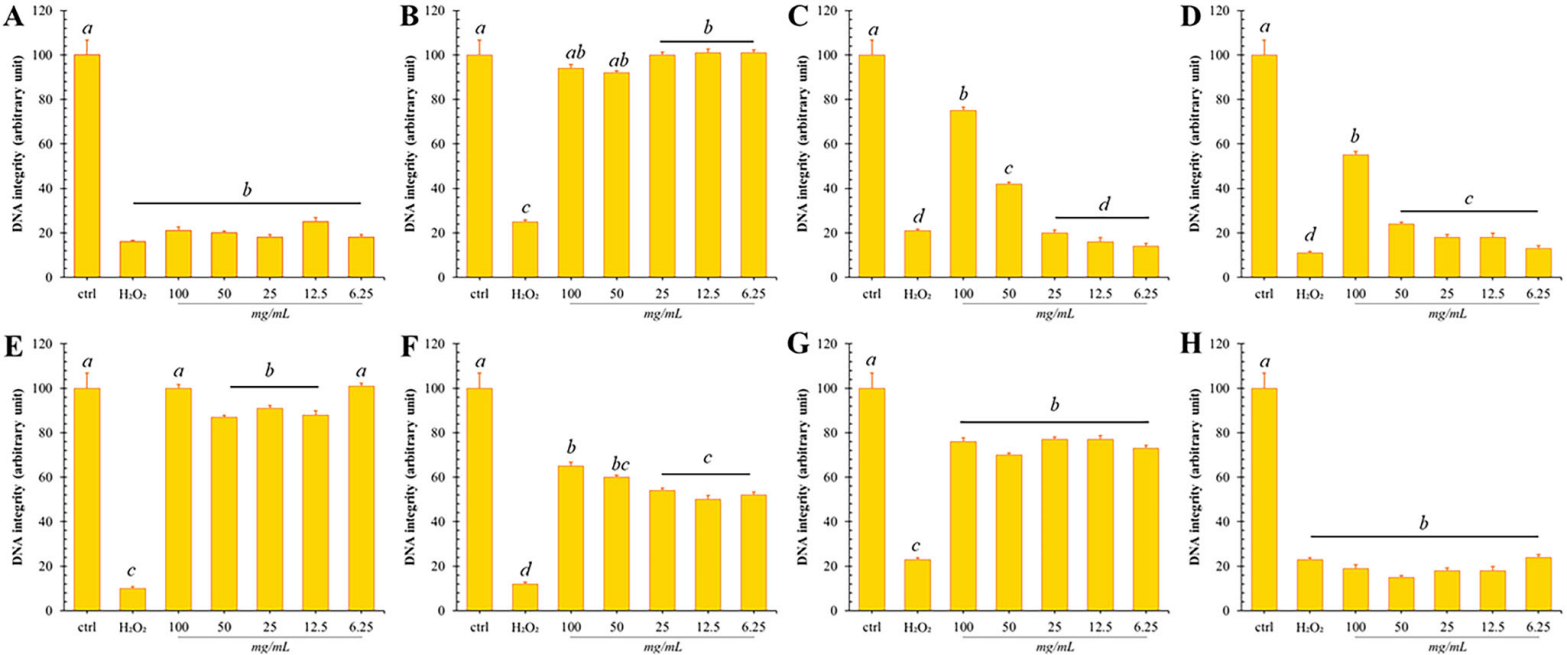
DNA Protective Potential of Pitaya Fruit Fractions. Effect of co‐incubation of different concentrations (100–6.25 mg mL^−1^) of the eight fractions (F1–F8, shown in panels A–H) on plasmid DNA integrity following exposure to 50 µM H_2_O_2_ and UV light. Control bars represent plasmid DNA without oxidative stress exposure, while the “H_2_O_2_” bars indicate plasmid DNA treated with 50 µM H_2_O_2_ and exposed to UV light for 30 min.

F2 fractions showed the best antioxidant capacities in almost all in‐solution and cellular assays (Table 1), including insult protection of oxidative damage (Figure 4B), making them particularly attractive from a bioactive point of view. In particular, F2 stands out with the lowest IC_50_ values in ABTS and DPPH assays, suggesting potent free radical scavenging activity (Table 1). This finding is supported by its high reducing activity in the FRAP assay and strong protection of DNA integrity from oxidative insult (Figure 4B). However, the recorded cellular antioxidant effect shows some discrepancy with previously recorded values, recording intermediate values. The chemical composition of F2 reveals a high concentration of betalains, including betanidine and neobetanine, which are known for their potent antioxidant activity. In addition, F2 is enriched with flavonoids, particularly rhamnetin‐arabinoside, apigenin‐glucuronide, and myricetin, which further amplify the antioxidant activity of the fraction. Consequently, the strong antioxidant capacity of F2 likely stems primarily from its betalain content; however, the contribution of co‐occurring flavonoids, including rhamnetin‐arabinoside, cannot be entirely excluded and may play a supporting role.

**TABLE 1:** Antioxidant properties of individual fractions from pitaya pulp extracts. ABTS and DPPH results are presented as IC_50_ values, indicating the amount of fraction required for 50% inhibition of radical oxidation. FRAP results are expressed as µmol of Trolox equivalents (TE) per gram of lyophilized fraction weight, while CAA_50_ is expressed as micromoles of TE per mL of cell culture medium. For each column, different lowercase letters indicate significant differences as assessed by Tukey's t‐test followed by post hoc analysis.

	Radical scavenging activity		Reducing activity	Cellular antioxidant activity
	*ABTS (IC_50_)*	*DPPH (IC_50_)*	*FRAP (µmol TE/g)*	*CAA_50_ (µmol TE/mL)*
**F1**	25.1779 ± 0.5988^f^	65.5412 ± 0.9874^g^	0.8512 ± 0.0012^e^	126.2 ± 12.4^g^
**F2**	0.2064 ± 0.0021^a^	0.4396 ± 0.0056^a^	316.3088 ± 1.839^a^	195.4 ± 7.1^d^
**F3**	6.9855 ± 0.0707^e^	6.9127 ± 0.184^c^	27.7544 ± 2.1476^c^	78.4 ± 9.37^c^
**F4**	6.8841 ± 0.0788^e^	25.0257 ± 0.788^f^	31.4051 ± 0.4486^c^	359.85 ± 4.41^f^
**F5**	2.0594 ± 0.0648^c^	5.696 ± 0.2172^c^	142.4446 ± 12.4012^b^	38.7 ± 7.27^b^
**F6**	6.005 ± 0.0368^d^	33.8937 ± 1.2323^e^	26.9186 ± 0.5828^c^	285.41 ± 8.89^e^
**F7**	0.4524 ± 0.0112^b^	3.4592 ± 0.1239^b^	38.8584 ± 1.8442^c^	21.3 ± 2.21^a^
**F8**	6.8449 ± 0.1425^e^	19.4358 ± 0.4314^d^	3.6341 ± 0.256^d^	215.58 ± 9.63^e^

Similarly, the F5 and F7 fractions also showed a good antioxidant profile, albeit with lower efficacy than F2. F7 showed good free radical scavenging activity but low reducing capacity, while F5 showed moderate levels according to all the models tested (Table 1). In contrast, assays evaluating antioxidant potential from a biological point of view showed that these fractions were instead the most active in terms of CAA_50_ and offered almost complete protection to DNA at all the ranges of the tested concentrations (Figure 4E,G).

On the other hand, the F1 fraction, which had been identified as having the poorest chemical profile in terms of either polyphenols or betalains by UV/Vis and HPLC detections, was also found to demonstrate the poorest antioxidant properties, as evidenced by its high IC_50_ values in the DPPH and ABTS assays, as well as the low FRAP values (Table 1). The data obtained from the solution assays correlated strongly with F1's lack of ability to protect DNA from oxidative insults, as illustrated in Figure 4A. However, when the same fraction was analyzed using a more biological assay, CAA, moderate antioxidant action was observed. The CAA assay allows the antioxidant capacity of an extract or compound to be evaluated independently of its intrinsic reducing or scavenging abilities. Considering that F1 is sparsely enriched with antioxidant molecules, the absence of protection from chemically generated ROS (as in the case of ABTS or DPPH), naturally occurring ROS (as in the case of H_2_O_2_ in the DNA protection assay), or oxidizing ions (as in the case of FRAP) can be easily justified (Wołosiak et al. 2022; Payne et al. 2013; Pinela et al. 2024). However, from the CAA results, it is plausible that these same molecules, while not exerting direct action against ROS, may exert antioxidant effects through alternative mechanisms. One possible explanation is that these molecules interact with specific proteins located on the outer surface of the membrane, which, when activated, trigger intracellular mechanisms that lead to the positive regulation of specific antioxidant genes such as SOD, CAT, GPX, or the increase of other cellular metabolites with antioxidant action (such as glutathione) (Gusti et al. 2021; Wu et al. 2004; Di Giacomo et al. 2023).

Placed in an intermediate position are all the other fractions, which demonstrate moderate radical scavenging and reducing activities, and poor CAA_50_ values (Table 1). These same fractions were also those demonstrating protective effects on DNA oxidation in a dose‐dependent manner, and provided about 50% of DNA protection at the highest doses.

## 4 Conclusion

This study provides a comprehensive characterization of the phytochemical profile and antioxidant properties of *Hylocereus hybridum* (pitaya) fruit and its fractions, underscoring the significant biological potential of this plant source. HPLC‐DAD‐MS/MS analysis definitively established the predominance of betalains, complemented by a substantial presence of flavonoids and other polyphenols within the fruit. The robust antioxidant capabilities of the pitaya extract were consistently confirmed through various in vitro assays, positioning the fruit as a competitive natural antioxidant source. Moreover, our chromatographic fractionation approach effectively linked specific chemical compositions to distinct biological activities. Fraction F2, notably rich in betacyanin‐related compounds, emerged as a particularly potent antioxidant in vitro and demonstrated strong protection against oxidative DNA damage. Interestingly, while fractions F5 and F7 exhibited more moderate in vitro antioxidant activity, they showed excellent cellular protection, suggesting the presence of bioactive compounds with specific, perhaps indirect, mechanisms of action within a cellular context. Conversely, fraction F1, characterized by a low concentration of antioxidant compounds, displayed limited efficacy, thereby reinforcing the direct correlation between the fruit's phytochemical composition and its observed biological functionality. These findings collectively advance our understanding of pitaya's health‐promoting attributes and highlight the importance of its diverse bioactive compounds. Overall, the results highlight the nutraceutical and pharmacological potential of pitaya fruit, both as a natural source of antioxidants and as a candidate for therapeutic applications, also providing valuable insights into pitaya's antioxidant properties. To fully elucidate its cellular mechanisms and support its therapeutic potential, future research should incorporate gene and protein expression analyses (e.g., SOD, CAT, GST) to understand how it modulates endogenous defense systems, and intestinal absorption studies (e.g., the Caco‐2 model) to assess bioavailability and in vivo relevance. Consequently, further studies are required to explore their bioavailability, efficacy in in vivo models, and potential applications in the food and pharmaceutical industries. The phytochemical diversity and strong antioxidant activity observed make pitaya fruit a promising element for the development of innovative strategies aimed at preventing oxidative stress and related diseases.

## Author Contributions

**Noemi Gatti**: conceptualization, investigation, validation, visualization. **Graziella Serio**: conceptualization, investigation, validation, methodology. **Jaime Morante‐carriel**: conceptualization, investigation, funding acquisition, methodology, validation, writing – original draft. **Pietro Deusebio**: conceptualization, investigation, methodology, validation, visualization, writing – original draft, formal analysis. **Giulia Conti**: conceptualization, investigation, writing – original draft, methodology, validation, visualization, formal analysis. **Campo Eva**: conceptualization, investigation, writing – original draft, methodology, software, formal analysis, data curation, supervision. **Moez Maghrebi**: writing – review and editing, visualization, conceptualization, methodology. **Carla Gentile**: conceptualization, investigation, funding acquisition, writing – original draft, writing – review and editing, visualization, validation, methodology, software, formal analysis, project administration, resources, supervision, data curation. **Giuseppe Mannino**: conceptualization, investigation, funding acquisition, writing – original draft, writing – review and editing, validation, methodology, visualization, software, formal analysis, project administration, resources, supervision, data curation.

## Conflicts of Interest

The authors declare no conflicts of interest.

## Supporting information




**Supplementary Table**: jfds70502‐sup‐0001‐TableS1.pdf

## Data Availability

All data supporting the findings of this study are available within the article. Raw data, including unprocessed gel images, are available from the corresponding author upon reasonable request.

## References

[jfds70502-bib-0001] Agraharam, G. , A. Girigoswami , and K. Girigoswami . 2022. “Myricetin: A Multifunctional Flavonol in Biomedicine.” Current Pharmacology Reports 8, no. 1: 48–61. 10.1007/s40495-021-00269-2. 35036292 PMC8743163

[jfds70502-bib-0002] Alfaro Jiménez, M. A. , A. Zugasti Cruz , S. Y. Silva Belmares , J. A. Ascacio Valdés , and C. A. Sierra Rivera . 2022. “Phytochemical and Biological Characterization of the Fractions of the Aqueous and Ethanolic Extracts of Parthenium Hysterophorus.” Separations 9: 359. 10.3390/separations9110359.

[jfds70502-bib-0003] Ali, F. , Rahul , F. Naz , S. Jyoti , and Y. H. Siddique . 2017. “Health Functionality of Apigenin: A Review.” International Journal of Food Properties 20, no. 6: 1197–1238. 10.1080/10942912.2016.1207188.

[jfds70502-bib-0004] Andonova, T. , Y. Muhovski , I. Slavov , et al. 2023. “Phenolic Profile, Antioxidant and DNA‐Protective Capacity, and Microscopic Characters of Ailanthus Altissima Aerial Substances.” Plants 12, no. 4: 920. 10.3390/plants12040920. 36840268 PMC9967504

[jfds70502-bib-0005] Bajpai, V. K. , R. Majumder , and J. G. Park . 2016. “Isolation and Purification of Plant Secondary Metabolites Using Column‐Chromatographic Technique.” Bangladesh Journal of Pharmacology 11, no. 4: 844–848. 10.3329/bjp.v11i4.28185.

[jfds70502-bib-0076] Belmonte‐Herrera, B. H. , J. A. Domínguez‐Avila , A. Wall‐Medrano , et al. 2022. “Lesser‐Consumed Tropical Fruits and Their By‐Products: Phytochemical Content and Their Antioxidant and Anti‐Inflammatory Potential.” Nutrients 14, no. 17: 3663. . 36079920 10.3390/nu14173663 PMC9460136

[jfds70502-bib-0006] Belmonte‐Herrera, B. H. , J. A. Domínguez‐Avila , A. Wall‐Medrano , et al. 2022. “Lesser‐Consumed Tropical Fruits and Their By‐Products: Phytochemical Content and Their Antioxidant and Anti‐Inflammatory Potential.” Nutrients 14, no. 17: 3663. 10.3390/nu14173663. 36079920 PMC9460136

[jfds70502-bib-0077] Benzie I. F. , and J. J. Strain . 1996. “The Ferric Reducing Ability of Plasma (FRAP) as a Measure of “Antioxidant Power”: The FRAP Assay.” Analytical Biochemistry 239, no. 1:70–76. . 8660627 10.1006/abio.1996.0292

[jfds70502-bib-0007] Benzie I. F. , and J. J. Strain . 1996. “The Ferric Reducing Ability of Plasma (FRAP) as a Measure of “Antioxidant Power”: The FRAP Assay.” Analytical Biochemistry 239, no. 1:70–76. 10.1006/abio.1996.0292. 8660627

[jfds70502-bib-0009] Brand‐Williams, W. , M. E. Cuvelier , and C. L. W. T. Berset . 1995. “Use of a Free Radical Method to Evaluate Antioxidant Activity.” LWT‐Food Science and Technology 28: 25–30. 10.1016/S0023-6438(95)80008-5.

[jfds70502-bib-0010] Calva‐Estrada, S. J. , M. Jiménez‐Fernández , and E. Lugo‐Cervantes . 2022. “Betalains and Their Applications in Food: The Current State of Processing, Stability and Future Opportunities in the Industry.” Food Chemistry: Molecular Sciences 4: 100089. 10.1016/j.fochms.2022.100089. 35415668 PMC8991513

[jfds70502-bib-0011] Campobenedetto, C. , C. Agliassa , G. Mannino , et al. 2021. “A Biostimulant Based on Seaweed (*Ascophyllum nodosum* and *Laminaria digitata*) and Yeast Extracts Mitigates Water Stress Effects on Tomato (*Solanum lycopersicum* L.).” Agriculture 11: 557. 10.3390/agriculture11060557.

[jfds70502-bib-0012] Campobenedetto, C. , G. Mannino , C. Agliassa , et al. 2020. “Transcriptome Analyses and Antioxidant Activity Profiling Reveal the Role of a Lignin‐Derived Biostimulant Seed Treatment in Enhancing Heat Stress Tolerance in Soybean.” Plants 9: 1308. 10.3390/plants9101308. 33023253 PMC7601093

[jfds70502-bib-0013] Cano A. , A. B. Maestre , J. Hernández‐Ruiz , and M. B. Arnao . 2023. “ABTS/TAC Methodology: Main Milestones and Recent Applications.” Processes 11, no. 1: 185. 10.3390/pr11010185.

[jfds70502-bib-0014] Chen, A. Y. , and Y. C. Chen . 2013. “A Review of the Dietary Flavonoid, Kaempferol on Human Health and Cancer Chemoprevention.” Food Chemistry 138, no. 4: 2099–2107. 10.1016/j.foodchem.2012.11.139. 23497863 PMC3601579

[jfds70502-bib-0015] Cheok A. , Y. Xu , Z. Zhang , P. W. Caton , and A. Rodriguez‐Mateos . 2022. “Betalain‐Rich Dragon Fruit (pitaya) Consumption Improves Vascular Function in Men and Women: A Double‐Blind, Randomized Controlled Crossover Trial.” American Journal of Clinical Nutrition 115, no. 5: 1418–1431. 10.1093/ajcn/nqab410. 35265960

[jfds70502-bib-0017] Chuang, S.‐Y. , Y.‐K. Lin , C.‐F. Lin , P.‐W. Wang , E.‐L. Chen , and J.‐Y. Fang . 2017. “Elucidating the Skin Delivery of Aglycone and Glycoside Flavonoids: How the Structures Affect Cutaneous Absorption.” Nutrients 9, no. 12: 1304. 10.3390/nu9121304. 29189718 PMC5748754

[jfds70502-bib-0018] Chunhakant, S. , and C. Chaicharoenpong . 2021. “Phytochemical Composition, Antioxidant and Antityrosinase Activities, and Quantification of (+)‐Dihydrokaempferol of Different Parts of Manilkara Zapota.” Indian Journal of Pharmaceutical Sciences 83, no. 6: 1144–1154. 10.36468/pharmaceutical-sciences.869.

[jfds70502-bib-0019] Collins, A. , P. Møller , G. Gajski , et al. 2023. “Measuring DNA Modifications With the Comet Assay: A Compendium of Protocols.” Nature Protocols 18, no. 3: 929–989. 10.1038/s41596-022-00754-y. 36707722 PMC10281087

[jfds70502-bib-0020] Corimayhua‐Silva, A. A. , C. Elías‐Peñafiel , T. Rojas‐Ayerve , A. Guevara‐Pérez , L. Farfán‐Rodríguez , and C. R. Encina‐Zelada . 2024. “Red Dragon Fruit Peels: Effect of Two Species Ratio and Particle Size on Fibre Quality and Its Application in Reduced‐Fat Alpaca‐Based Sausages.” Foods 13: 386. 10.3390/foods13030386. 38338524 PMC10855916

[jfds70502-bib-0021] Cortat B. , C. C. Garcia , A. Quinet , A. P. Schuch , K. M. de Lima‐Bessa , and C. F. Menck . 2013. “The Relative Roles of DNA Damage Induced by UVA Irradiation in Human Cells.” Photochemical & Photobiological Sciences 12, no. 8:1483–1495. 10.1039/c3pp50023c. 23824260

[jfds70502-bib-0023] Di Giacomo, C. , G. A. Malfa , B. Tomasello , S. Bianchi , and R. Acquaviva . 2023. “Natural Compounds and Glutathione: Beyond Mere Antioxidants.” Antioxidants 12, no. 7: 1445. 10.3390/antiox12071445. 37507985 PMC10376414

[jfds70502-bib-0024] Di Minno A. , L. Turnu , B. Porro , et al. 2016. “8‐Hydroxy‐2‐Deoxyguanosine Levels and Cardiovascular Disease: A Systematic Review and Meta‐Analysis of the Literature.” Antioxid Redox Signaling 24, no. 10: 548–555. 10.1089/ars.2015.6508. PMC4827317 26650622

[jfds70502-bib-0025] dos Santos Souza, E. J. , K. W. Fomba , M. van Pinxteren , N. Deabji , and H. Herrmann . 2024. “Strong Synergistic and Antagonistic Effects of Quinones and Metal Ions in Oxidative Potential (OP) Determination by Ascorbic Acid (AA) Assays.” Journal of Hazardous Materials 478: 135599. 10.1016/j.jhazmat.2024.135599. 39180997

[jfds70502-bib-0026] Floegel, A. , D. O. Kim , S. J. Chung , S. I. Koo , and O. K. Chun . 2011. “Comparison of ABTS/DPPH Assays to Measure Antioxidant Capacity in Popular Antioxidant‐Rich US Foods.” Journal of Food Composition and Analysis 24, no. 7: 1043–1048. 10.1016/j.jfca.2011.01.008.

[jfds70502-bib-0027] Guo, C. , J. Yang , J. Wei , Y. Li , J. Xu , and Y. Jiang . 2003. “Antioxidant Activities of Peel, Pulp and Seed Fractions of Common Fruits as Determined by FRAP Assay.” Nutrition Research 23, no. 12: 1719–1726. 10.1016/j.nutres.2003.08.005.

[jfds70502-bib-0028] Gusti A. M. T. , S. Y. Qusti , E. M. Alshammari , E. A. Toraih , and M. S. Fawzy . 2021. “Antioxidants‐Related Superoxide Dismutase (SOD), Catalase (CAT), Glutathione Peroxidase (GPX), Glutathione‐S‐Transferase (GST), and Nitric Oxide Synthase (NOS) Gene Variants Analysis in an Obese Population: A Preliminary Case‐Control Study.” Antioxidants 10, no. 4: 595. 10.3390/antiox10040595. 33924357 PMC8070436

[jfds70502-bib-0029] Huang, Y. , M. A. Brennan , S. Kasapis , S. J. Richardson , and C. S. Brennan . 2021. “Maturation Process, Nutritional Profile, Bioactivities and Utilisation in Food Products of Red Pitaya Fruits: A Review.” Foods 10, no. 11: 2862. 10.3390/foods10112862. 34829143 PMC8618204

[jfds70502-bib-0030] Kellett, M. E. , P. Greenspan , and R. B. Pegg . 2018. “Modification of the Cellular Antioxidant Activity (CAA) Assay to Study Phenolic Antioxidants in a Caco‐2 Cell Line.” Food Chemistry 244, no. 706: 359–363. 10.1016/j.foodchem.2017.10.035. 29120793

[jfds70502-bib-0031] Lee H. , M. Krishnan , M. Kim , Y. K. Yoon , and Y. Kim . 2022. “Rhamnetin, a Natural Flavonoid, Ameliorates Organ Damage in a Mouse Model of Carbapenem‐Resistant Acinetobacter Baumannii‐Induced Sepsis.” International Journal of Molecular Sciences 23, no. 21: 12895. 10.3390/ijms232112895. 36361685 PMC9656386

[jfds70502-bib-0032] Liu, W. , Y. Feng , S. Yu , et al. 2021. “The Flavonoid Biosynthesis Network in Plants.” International Journal of Molecular Sciences 22: 12824. 10.3390/ijms222312824. 34884627 PMC8657439

[jfds70502-bib-0078] Lopez Martinez, C. R. , R. G. Mateos , C. G. Vázquez , and J. S. Castellanos . 2015. “Antioxidant Components and Nutritional Quality of 15 Genotypes of Xoconostle (Opuntia spp.).” Journal of the Professional Association for Cactus Development 17: 33–49.

[jfds70502-bib-0035] Madadi E. , S. Mazloum‐Ravasan , J. S. Yu , J. W. Ha , H. Hamishehkar , and K. H. Kim . 2020. “Therapeutic Application of Betalains: A Review.” Plants 9, no. 9: 1219. 10.3390/plants9091219. 32957510 PMC7569795

[jfds70502-bib-0079] Mannino, G. , G. Serio , C. M. Bertea , R. Chiarelli , A. Lauria , and C. Gentile 2022. “Phytochemical Profile and Antioxidant Properties of the Edible and Non‐edible Portions of Black Sapote (Diospyros digyna Jacq.).” Food Chemistry 380: 132137. 10.1016/j.foodchem.2022.132137. 35093655

[jfds70502-bib-0036] Mannino, G. , G. Chinigò , G. Serio , et al. 2021. “Proanthocyanidins and Where to Find Them: A Meta‐Analytic Approach to Investigate Their Chemistry, Biosynthesis, Distribution, and Effect on Human Health.” Antioxidants 10, no. 8: 1229. 10.3390/antiox10081229. 34439477 PMC8389005

[jfds70502-bib-0037] Mannino, G. , C. Gentile , and M. E. Maffei . 2019. “Chemical Partitioning and DNA Fingerprinting of Some Pistachio (*Pistacia vera* L.) Varieties of Different Geographical Origin.” Phytochemistry 160: 40–47. 10.1016/j.phytochem.2019.01.010. 30690343

[jfds70502-bib-0038] Miraj, S. 2016. “Chemistry and Pharmacological Effect of Beta Vulgaris.” *A Systematic Review* 8: 404–409.

[jfds70502-bib-0039] Mohammed, M. S. , W. J. A. Osman , E. A. E. Garelnabi , et al. 2014. “Secondary Metabolites as Anti‐Inflammatory Agents.” The Journal of Phytopharmacology 3, no. 4: 275–285. 10.31254/phyto.2014.3409.

[jfds70502-bib-0040] Morales, P. , L. Barros , E. Ramírez‐Moreno , C. Santos‐Buelga , and I. C. Ferreira . 2015. “Xoconostle Fruit (Opuntia matudae Scheinvar cv. Rosa) By‐Products as Potential Functional Ingredients.” Food Chemistry 185: 289–297. 10.1016/j.foodchem.2015.04.012. 25952871

[jfds70502-bib-0041] Munteanu I. G. , and C. Apetrei . 2021. “Analytical Methods Used in Determining Antioxidant Activity: A Review.” International Journal of Molecular Sciences 22, no. 7: 3380. 10.3390/ijms22073380. 33806141 PMC8037236

[jfds70502-bib-0042] Nishikito, D. F. , A. C. A. Borges , L. F. Laurindo , et al. 2023. “Anti‐Inflammatory, Antioxidant, and Other Health Effects of Dragon Fruit and Potential Delivery Systems for Its Bioactive Compounds.” Pharmaceutics 15: 159. 10.3390/pharmaceutics15010159. 36678789 PMC9861186

[jfds70502-bib-0048] Pérez‐Jiménez, J. , V. Neveu , F. Vos , et al. 2020. “Identification of the 100 Richest Dietary Sources of Polyphenols: An Application of the Phenol‐Explorer Database.” European Journal of Clinical Nutrition 64, no. Suppl 3: S112–S120. 10.1038/ejcn.2010.221. 21045839

[jfds70502-bib-0044] Paśko, P. , A. Galanty , P. Zagrodzki , et al. 2021. “Bioactivity and Cytotoxicity of Different Species of Pitaya Fruits—A Comparative Study With Advanced Chemometric Analysis.” Food Bioscience 40: 100888. 10.1016/j.fbio.2021.100888.

[jfds70502-bib-0043] Pandey, K. B. , and S. I. Rizvi . 2009. “Plant Polyphenols as Dietary Antioxidants in Human Health and Disease.” Oxidative Medicine and Cellular Longevity 2, no. 5: 270–278. 10.4161/oxim.2.5.9498. 20716914 PMC2835915

[jfds70502-bib-0045] Payne, A. C. , A. Mazzer , G. J. J. Clarkson , and G. Taylor . 2013. “Antioxidant Assays—Consistent Findings From FRAP and ORAC Reveal a Negative Impact of Organic Cultivation on Antioxidant Potential in Spinach but Not Watercress or Rocket Leaves.” Food Science & Nutrition 1, no. 6: 439–444. 10.1002/fsn3.71. 24804054 PMC3951540

[jfds70502-bib-0049] Pinela J. , M. I. Dias , C. Pereira , and J. I. Alonso‐Esteban . 2024. “Antioxidant Activity of Foods and Natural Products.” Molecules 29, no. 8: 1814. 10.3390/molecules29081814. 38675634 PMC11054945

[jfds70502-bib-0050] Polturak, G. , and A. Aharoni . 2018. ““La Vie En Rose”: Biosynthesis, Sources, and Applications of Betalain Pigments.” Molecular Plant 11, no. 1: 7–22. 10.1016/j.molp.2017.10.008. 29081360

[jfds70502-bib-0052] Rauf, A. , M. Imran , T. Abu‐Izneid , et al. 2019. “Proanthocyanidins: A Comprehensive Review.” Biomedicine & Pharmacotherapy 116: 108999. 10.1016/j.biopha.2019.108999. 31146109

[jfds70502-bib-0053] Ravichandran, G. , D. K. Lakshmanan , S. Murugesan , A. Elangovan , N. S. Rajasekaran , and S. Thilagar . 2021. “Attenuation of Protein Glycation by Functional Polyphenolics of Dragon Fruit (*Hylocereus polyrhizus*); An In Vitro and In Silico Evaluation.” Food Research International 140: 110081. 10.1016/j.foodres.2020.110081. 33648300

[jfds70502-bib-0054] Re R. , N. Pellegrini , A. Proteggente , A. Pannala , M. Yang , and C. Rice‐Evans . 1999. “Antioxidant Activity Applying an Improved ABTS Radical Cation Decolorization Assay.” Free Radical Biology & Medicine 26, no. 9‐10: 1231–1237. 10.1016/S0891-5849(98)00315-3. 10381194

[jfds70502-bib-0055] Sakuta M. , A. Tanaka , K. Iwase , et al. 2021. “Anthocyanin Synthesis Potential in Betalain‐Producing Caryophyllales Plants.” Journal of Plant Research 134, no. 6: 1335–1349. 10.1007/s10265-021-01341-0. 34477986 PMC8930957

[jfds70502-bib-0056] Salehi, B. , A. Venditti , M. Sharifi‐Rad , et al. 2019. “The Therapeutic Potential of Apigenin.” International Journal of Molecular Sciences 20, no. 6: 1305. 10.3390/ijms20061305. 30875872 PMC6472148

[jfds70502-bib-0057] Sasidharan S. , Y. Chen , D. Saravanan , K. M. Sundram , and L. Yoga Latha . 2011. “Extraction, Isolation and Characterization of Bioactive Compounds From Plants' Extracts.” African Journal of Traditional, Complementary and Alternative Medicines 8, no. 1: 1–10. PMC3218439 22238476

[jfds70502-bib-0058] Schaub, J. , A. Zielesny , C. Steinbeck , and M. Sorokina . 2021. “Description and Analysis of Glycosidic Residues in the Largest Open Natural Products Database.” Biomolecules 11, no. 4: 486. 10.3390/biom11040486. 33804966 PMC8063959

[jfds70502-bib-0059] Sgroi, F. , F. Modica , C. Sciortino , and F. Fusté‐Forné . 2023. “Changing Crops, Changing Diets: Consumers' Purchase Intention of Sicilian Tropical Fruits.” International Journal of Gastronomy and Food Science 33: 100777. 10.1016/j.ijgfs.2023.100777.

[jfds70502-bib-0060] Shah, K. , J. Chen , J. Chen , and Y. Qin . 2023. “Pitaya Nutrition, Biology, and Biotechnology: A Review.” International Journal of Molecular Sciences 24, no. 18: 1–28. 10.3390/ijms241813986. PMC10530492 37762287

[jfds70502-bib-0061] Shraim, A. M. , T. A. Ahmed , M. M. Rahman , and Y. M. Hijji . 2021. “Determination of Total Flavonoid Content by Aluminum Chloride Assay: A Critical Evaluation.” Lwt 150: 111932. 10.1016/j.lwt.2021.111932.

[jfds70502-bib-0062] Sonter, S. , S. Mishra , M. K. Dwivedi , and P. K. Singh . 2021. “Chemical Profiling, In Vitro Antioxidant, Membrane Stabilizing and Antimicrobial Properties of Wild Growing *Murraya paniculata* From Amarkantak (M.P.).” Scientific Reports 11, no. 1: 1–15. 10.1038/s41598-021-87404-7. 33963198 PMC8105327

[jfds70502-bib-0063] Stintzing, F. C. , K. M. Herbach , M. R. Mosshammer , et al. 2005. “Color, Betalain Pattern, and Antioxidant Properties of Cactus Pear (Opuntia spp.) Clones.” Journal of Agricultural and Food Chemistry 53, no. 2: 442–451. 10.1021/jf048751y. 15656686

[jfds70502-bib-0064] Suárez‐Cáceres, G. P. , M. Malia‐Torrejón , L. Pérez‐Urrestarazu , J. A. Gross , and V. M. Fernández‐Cabanás . 2024. “Evaluation of Rooting and Growth of Pitaya (Hylocereus spp.) Cuttings in Soilless Production: Comparison of Hydroponic vs. Aquaponic Systems.” Cogent Food & Agriculture 10, no. 1: 2355298. 10.1080/23311932.2024.2355298.

[jfds70502-bib-0065] Thwe P. N. , K. Y. Yeong , and W. S. Choo . 2023. “Anti‐Amyloid β Aggregation Activity and Cell Viability Effect of Betacyanins From Red Pitahaya (*Hylocereus polyrhizus*) for Alzheimer's Disease.” Plant Foods for Human Nutrition 78, no. 3: 613–619. 10.1007/s11130-023-01081-7. 37466824

[jfds70502-bib-0066] Trindade, A. R. , P. Paiva , V. Lacerda , N. Marques , L. Neto , and A. Duarte . 2023. “Pitaya as a New Alternative Crop for Iberian Peninsula: Biology and Edaphoclimatic Requirements.” Plants 12: 3212. 10.3390/plants12183212. 37765376 PMC10537634

[jfds70502-bib-0080] Tsai, Y. , C. G. Lin , W. L. Chen , et al. 2019. “Evaluation of the Antioxidant and Wound‐healing Properties of Extracts From Different Parts of Hylocereus Polyrhizus.”. Agronomy 9, no. 1: 27. 10.3390/agronomy9010027.

[jfds70502-bib-0068] Valverde M. , J. Lozano‐Salgado , P. Fortini , M. A. Rodriguez‐Sastre , E. Rojas , and E. Dogliotti . 2018. “Hydrogen Peroxide‐Induced DNA Damage and Repair Through the Differentiation of Human Adipose‐Derived Mesenchymal Stem Cells.” Stem Cells International 2018: 1615497. 10.1155/2018/1615497. 30405718 PMC6199883

[jfds70502-bib-0069] Vieira Teixeira da Silva, D. , D. dos Santos Baião , F. de Oliveira Silva , et al. 2019. “Betanin, a Natural Food Additive: Stability, Bioavailability, Antioxidant and Preservative Ability Assessments.” Molecules 24, no. 3: 458. 10.3390/molecules24030458. 30696032 PMC6384587

[jfds70502-bib-0070] Wijesinghe, V. N. , and W. S. Choo . 2022. “Antimicrobial Betalains.” Journal of Applied Microbiology 133, no. 6: 3347–3367. 10.1111/jam.15798. 36036373 PMC9826318

[jfds70502-bib-0072] Wołosiak, R. , B. Drużyńska , D. Derewiaka , et al. 2022. “Verification of the Conditions for Determination of Antioxidant Activity by Abts and Dpph Assays—A Practical Approach.” Molecules 27, no. 1: 50. 10.3390/molecules27010050. PMC8747050 35011274

[jfds70502-bib-0071] Wolfe, K. L. , and R. H. Liu . 2007. “Cellular Antioxidant Activity (CAA) Assay for Assessing Antioxidants, Foods, and Dietary Supplements.” Journal of Agricultural and Food Chemistry 55, no. 22: 8896–8907. 10.1021/jf0715166. 17902627

[jfds70502-bib-0073] Wu, G. , J. R. Lupton , N. D. Turner , Y. Z. Fang , and S. Yang . 2004. “Glutathione Metabolism and Its Implications for Health.” The Journal of Nutrition 134, no. 3: 489–492. 14988435 10.1093/jn/134.3.489

[jfds70502-bib-0074] Wybraniec S. , P. Stalica , G. Jerz , et al. 2009. “Separation of Polar Betalain Pigments From Cacti Fruits of Hylocereus Polyrhizus by Ion‐Pair High‐Speed Countercurrent Chromatography.” Journal of Chromatography A 1216, no. 41: 6890–6899. 10.1016/j.chroma.2009.08.035. 19732900

[jfds70502-bib-0075] Yu, S. M. , S. J. Kim , Y. C. Yoon , and J. H. Kim . 2021. “Development and Application of a Chemical Profiling Method for the Assessment of the Quality and Consistency of the Pelargonium Sidoides Extract.” Journal of Analytical Science and Technology 12, no. 1: 46. 10.1186/s40543-021-00297-z.

