# Supplementary material for: Phytochemical and Antioxidant Profile of Pitaya (Hylocereus hybridum) Fruits: Elucidation Through Chemical Fractionation
Source: J Food Sci. 2025 Aug 22;90(8):e70502. doi: 10.1111/1750-3841.70502 (PMC12371859; doi:10.1111/1750-3841.70502)
Supplement: Supplementary file 1 — Supplementary Table: jfds70502‐sup‐0001‐TableS1.pdf [file JFDS-90-0-s001.pdf]

Table S1: Spectral and mass spectrometric data for the observed compounds, including their maximum absorbance wavelengths (λ max), chemical structures, molecular weights (MW), m/z values, and characteristic MS/MS fragmentation ions

Compound(s)	***λ***	***Chemical Structure***	***MW***	***m/z***	***MS/MS***
betalamic acid	424	$C_9H_9NO_5$	211.17	210	
dihydrokaempferol	260; 280; 320	$C_{15}H_{12}O_6$	288.25	287	259, 125
quercetin	260; 280; 320	$C_{15}H_{10}O_7$	302.24	301	179, 151, 123
indicaxanthin	485	$C_{14}H_{16}N_2O_6$	308.29	309	291, 273, 245
hamnetin	260; 280; 320	$C_{15}H_{10}O_7$	302.24	301	179, 151, 123
myricetin	260; 280; 320	$C_{15}H_{10}O_8$	318.24	317	179, 151, 123
dihydromyricetin	260; 280; 320	$C_{15}H_{12}O_8$	320.25	319	193, 125
dihydrodecarboxy-neobetanidin	480; 520	$C_{13}H_{14}N_2O_5$	278.26	279	261, 233, 215
decarboxy-neobetanidin	480; 520	$C_{14}H_{14}N_2O_6$	292.27	293	275, 247, 229
betanidin	480; 520	$C_{17}H_{17}N_2O_4$	308.34	309	291, 273, 255
apigenin-rhamn	260; 280; 320	$C_{21}H_{20}O_9$	432.38	431	269, 146
kaempferol-ara	260; 280; 320	$C_{20}H_{18}O_8$	418.35	417	285, 132
dihydrokaempferol-ara	260; 280; 320	$C_{20}H_{20}O_9$	420.37	419	287, 132
apigenin-glucoside	260; 280; 320	$C_{21}H_{22}O_{10}$	448.4	447	269, 162
naringenin-glucoside	260; 280; 320	$C_{21}H_{22}O_{10}$	448.4	447	271, 162
apigenin-glucuronide	260; 280; 320	$C_{21}H_{18}O_{13}$	462.36	461	269, 176
kaempferol-glucoside	260; 280; 320	$C_{21}H_{20}O_{11}$	448.38	447	285, 162
dihydrokaempferol-glucoside	260; 280; 320	$C_{21}H_{22}O_{11}$	450.4	449	287, 162
dihydromyricetin-ara	260; 280; 320	$C_{21}H_{22}O_{12}$	466.4	465	319, 132
kaempferol-glucuronide	260; 280; 320	$C_{21}H_{18}O_{13}$	462.36	461	285, 176
quercetin-glucoside	260; 280; 320	$C_{21}H_{20}O_{12}$	464.38	463	301, 162
dihydroquercetin-glucoside	260; 280; 320	$C_{21}H_{22}O_{12}$	466.4	465	303, 162
hamnetin-glucoside	260; 280; 320	$C_{21}H_{20}O_{12}$	478.4	477	315, 162
myricetin-glucoside	260; 280; 320	$C_{21}H_{20}O_{13}$	480.38	479	317, 162
dihydromyricetin-glucoside	260; 280; 320	$C_{21}H_{22}O_{13}$	482.4	481	319, 162
hamnetin-glucuronide	260; 280; 320	$C_{21}H_{18}O_{14}$	494.36	493	315, 176
myricetin-glucuronide	260; 280; 320	$C_{21}H_{18}O_{15}$	496.36	495	317, 176
dihydromyricetin-glucuronide	260; 280; 320	$C_{21}H_{20}O_{15}$	498.38	497	319, 176
glu-decarboxy-neobetanidin	480; 520	$C_{20}H_{24}N_2O_{11}$	436.43	437	275, 257, 229
glu-dihydrodecarboxy-neobetanidin	480; 520	$C_{20}H_{26}N_2O_{11}$	438.45	439	277, 259, 231
isobetanin	480; 520	$C_{17}H_{17}N_2O_4$	308.34	309	291, 263, 24
apigenin-samb	260; 280; 320	$C_{21}H_{20}O_{10}$	432.38	431	269, 146
naringenin-samb	260; 280; 320	$C_{21}H_{22}O_{10}$	434.4	433	271, 146
apigenin-rut	260; 280; 320	$C_{27}H_{30}O_{14}$	594.52	593	269, 146
naringenin-rut	260; 280; 320	$C_{27}H_{32}O_{14}$	596.54	595	271, 146
dihydrokaempferol-samb	260; 280; 320	$C_{21}H_{22}O_9$	418.38	417	287, 146
kaempferol-rut	260; 280; 320	$C_{27}H_{30}O_{15}$	610.52	609	285, 146
dihydrokaempferol-rut	260; 280; 320	$C_{27}H_{32}O_{15}$	612.54	611	287, 146
dihydroquercetin-samb	260; 280; 320	$C_{21}H_{22}O_{11}$	434.39	433	303, 146
quercetin-rut	260; 280; 320	$C_{27}H_{30}O_{16}$	626.52	625	301, 146
dihydroquercetin-rut	260; 280; 320	$C_{27}H_{32}O_{16}$	628.54	627	303, 146
myricetin-rut	260; 280; 320	$C_{27}H_{30}O_{17}$	642.52	641	317, 146
dihydroquercetin-diglu	260; 280; 320	$C_{27}H_{34}O_{16}$	626.56	625	303, 324
phyllocactin	480; 520	$C_{21}H_{28}N_2O_{13}$	480.46	481	319, 301, 273
hamnetin-diglu	260; 280; 320	$C_{27}H_{32}O_{14}$	594.56	593	315, 324
myricetin-diglu	260; 280; 320	$C_{27}H_{32}O_{15}$	610.52	609	317, 324
dihydromyricetin-diglu	260; 280; 320	$C_{27}H_{34}O_{15}$	612.56	611	319, 324
Betanidin-5-O-(6'-O-3-hydroxy-3-methyl-glutaryl)-β-glucoside	480; 520	$C_{24}H_{29}N_2O_{15}$	533.5	534	390, 372, 308, 290
